# Requirements for mHealth and Augmented Reality Apps for Patient Education Regarding Colorectal Cancer Surgery: Focus Group Study

**DOI:** 10.2196/75972

**Published:** 2026-02-26

**Authors:** Steffen Busse, Verena Uslar, Sebastian Fudickar, Veysel Ödemis, Sandra Hellmers, Anja U Bräuer, Andreas Hein, Dirk Weyhe

**Affiliations:** 1 Assistance Systems and Medical Device Technology, Department of Health Services Research, School VI - Medicine and Health Sciences, Carl von Ossietzky Universität Oldenburg, Ammerländer Heerstraße 114-118, Oldenburg, 26129, Germany, 49 441-798 ext 2849; 2 University Clinic for Visceral Surgery, Department for Human Medicine, School VI - Medicine and Health Sciences, Carl von Ossietzky Universität Oldenburg, Oldenburg, Germany; 3 Institute of Medical Biometry and Statistics, Section for Clinical Research IT, University of Lübeck, Lübeck, Germany; 4 Fraunhofer Research Institution for Individualized and Cell-Based Medical Engineering, Lübeck, Germany; 5 Research Group Anatomy, Department for Human Medicine, School VI - Medicine and Health Sciences, Carl von Ossietzky Universität Oldenburg, Oldenburg, Germany

**Keywords:** informed consent, patient education, augmented reality, mixed reality, mHealth, smartphone, tablet, qualitative research, colorectal cancer

## Abstract

**Background:**

The purpose of preoperative informed consent is to provide patients with comprehensive information about their treatment, including risks and alternatives, to enable informed decision-making. However, studies have shown that patients are often unable to understand or remember important information. Mobile health (mHealth) and augmented reality (AR) apps have been identified as promising solutions to improve patient education and knowledge retention.

**Objective:**

This study aims to identify the essential requirements for an mHealth app to support informed decision-making for patients with colorectal cancer, with a specific focus on the potential of AR for visualization. This research explores the patient and physician perspectives on these requirements, particularly regarding information delivery and visualization to guide app design.

**Methods:**

A qualitative focus group study was conducted with groups of mostly patients with colorectal cancer and a physician’s group. Topics related to patient education were discussed, guided by a semistructured interview guide covering personal experience; information content; context of use; and acceptance and presentation of content, which included presenting various visualizations in 2D, 3D, and AR. The interviews were transcribed and analyzed using qualitative content analysis.

**Results:**

We conducted 4 focus groups with patients (n=23) and 1 focus group with physicians (n=7), for a total of 30 participants. Relevant informational content for the app and its presentation was identified. Patients consistently expressed a desire for personalized, detailed, and visual information about their condition and treatment tailored to their specific case throughout the treatment journey, so they could prepare for the informed consent discussion after diagnosis, prepare for treatment, access guidance and track progress during hospitalization, and access information and resources during recovery after treatment. Patients demonstrated a strong preference for interactive 3D visualizations, while physicians favored simpler 2D images that could be easily integrated into their existing workflow. AR visualizations were seen as a potential tool to provide a general overview of anatomy and surgical approaches but more as a novelty feature and a supplement to more traditional visualizations.

**Conclusions:**

An ideal patient education app combines comprehensive content with interactive, customizable visualizations like 3D models and AR and should be accessible throughout a patient’s treatment journey. This study highlights the need for a patient-centered design that balances detailed information with ease of understanding and considering different preferences for visualization modalities and levels of detail.

## Introduction

The purpose of preoperative informed consent is to provide the patient with a comprehensive explanation of the significance and scope of the proposed treatment, including treatment alternatives and the associated risks to enable them to make an informed decision [1]. Patient empowerment through education and informed decision-making has been shown to improve perioperative quality of care and increase patient satisfaction and compliance [2-4]. Physicians are often constrained by limited time, while the length of the conversation is the primary factor affecting comprehension [5]. In addition to the informed consent discussion, information sheets are provided to convey the information from the consultation. However, analyses have indicated that the text and images used in these sheets are unsuitable for supporting an informed decision [6]. Despite signing consent, most patients are not able to recall explanations about risks or alternative treatment options [7].

As colorectal cancer is the third most common form of cancer [8], it is essential to explore the challenges patients face in retaining knowledge about their condition. The experiences and perceptions of patients with colorectal cancer emphasize the need for individualized information that is tailored to their unique circumstances [9]. Mobile health (mHealth) refers to the use of mobile devices, such as smartphones and tablets, to support medical and public health practice [10], including the use of mobile apps for diagnostics and clinical decision-making, behavior change interventions, digital therapeutics, and apps that deliver disease-related education [11]. The increasing adoption of mHealth technologies offers a promising solution to improve patient education and address the need for personalized information [11,12]. mHealth platforms like apps for smartphones and tablets can provide patients with timely, relevant, and accessible information, leading to increased knowledge for patients compared with traditional educational practices [13,14].

It has been shown that the use of augmented reality (AR) apps—specifically displaying 3D models on a tablet in an AR setting by superimposing the 3D anatomical object onto the tablet’s camera video image, as if it were in the real world—as a supplement to traditional patient information leads to a better understanding and greater retention of the knowledge compared with 2D representations [15]. The use of AR in patient education also resulted in increased patient satisfaction and better health outcomes, as it has the potential to make learning more engaging and reduce cognitive load [16].

One form of AR technology is AR mirror systems, which use a mirror-like interface to display virtual information on top of a user’s camera image. In the context of anatomy education, AR mirrors have the potential to enhance learning by providing interactive 3D models of the human body [17-19]. So far, it has only been used in anatomy teaching for medical students.

This study used a qualitative approach using focus group interviews with patients and physicians to identify the essential requirements for an mHealth smartphone or tablet app to support informed decision-making for patients with colorectal cancer, with a specific focus on the potential of AR mirrors for visualization. This research explored the patient and physician perspectives on these requirements, particularly regarding information delivery and visualization to guide app design.

## Methods

### Ethical Considerations

The study was approved by the ethical review board of the Carl von Ossietzky Universität Oldenburg (registration number: 2021‐147) and was conducted in accordance with the Declaration of Helsinki [20]. All participants provided written informed consent prior to participation, and no compensation was offered. The study data, including video transcripts and questionnaires, are deidentified. Video recordings were transcribed, with participant names replaced with abbreviations, leaving only deidentified transcripts after deletion of the video.

### Methodological Approach

We used the human-centered design process, which is an iterative process focused on understanding user needs for interactive systems [21]. We used qualitative research with focus groups to achieve the initial steps of understanding and defining the context of use and defining user requirements. A focus group is a carefully planned discussion where participants share and comment on a defined topic [22] from personal experience and leads to rich insights about perceptions, thoughts, feelings, and impressions [23,24]. A moderator facilitates open, uninhibited dialog by using a nonprescriptive, semistructured approach supplementing prepared questions while also being receptive to relevant issues raised by participants [23]. The focus groups were conducted between July 2022 and September 2023.

### Data Collection

The focus groups were designed as semistructured group discussions, with an interview guide provided to assist the moderators and ensure consistency across discussions. All focus groups followed the same interview guide, with items adapted to physicians and patients to allow patients to speak from experience and physicians to report information they considered most important for patients. The individual items consisted of specific questions as well as stimuli in the form of statements or illustrations, which were intended to encourage a natural discussion [25]. See Multimedia Appendix 1 for the full interview guide. The interview guide addressed the following topics: personal experience with patient education (how the patient education took place and the typical procedure), information content topics: (content that is helpful and important from participants’ experience and gaps in patient education content), presentation of content (how the treatment is visualized), context of use (in which environment and with which people an app would be used), and acceptance (general opinion about the use of an app for patient education and factors that could lead to higher use).

As an incentive, the participants were presented with different types of visualization. The *Visualizations and AR Prototype* section provides a detailed description of the visualizations. The visualizations were presented sequentially, starting with the 2D images, progressing to the 3D model, and concluding with the AR mirror visualization developed for this study. Participants were given time to examine each visualization and discuss their impressions. The moderators asked questions about the possible advantages and disadvantages of each option.

To foster an open and uninhibited atmosphere, participants were encouraged to freely express their thoughts, with the assurance that there were no incorrect responses. The questions and stimuli were presented via the use of a projector. Following the discussion, all participants were requested to complete a questionnaire regarding demographic data (age, gender) and a standardized questionnaire for technical commitment by Neyer et al [26]. This 12-item questionnaire uses a 5-point Likert scale to assess the willingness to use technology in terms of technology acceptance, competence, and control. Smartphone and tablet use were also assessed via self-reporting using a 5-point Likert scale ranging from *never* to *always* (see Multimedia Appendix 2).

The focus groups were conducted in various locations, including a meeting room at a hospital (group 1), a meeting room at the university (groups 2‐4), and a seminar room at a rehabilitation hospital (group 5). The focus groups were conducted by the same two moderators, except for group 3, which was conducted by only one of the moderators. Audio and video recordings were made of all focus groups. The video recordings were used solely to more accurately assign the audio track to the participants.

### Visualizations and AR Prototype

**Figure 1.**
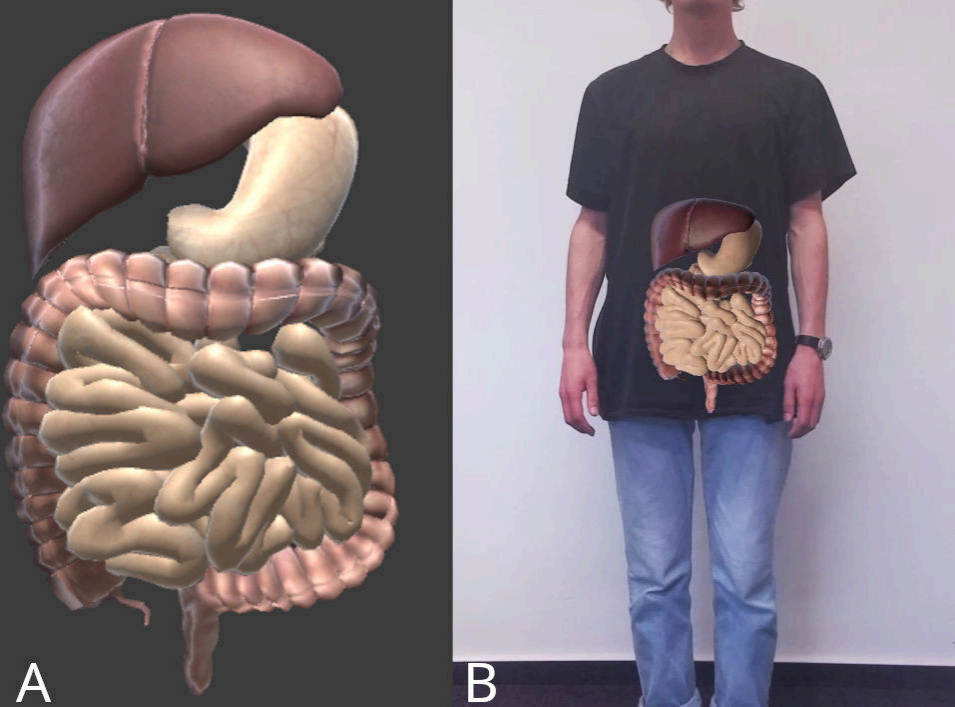
(A) Rotatable 3D model of the digestive system (liver, stomach, small intestine, large intestine) and (B) augmented reality (AR) 3D model projected on a body.

The visualizations used in the focus groups consisted of colored, detailed drawings of only the colon [27] and the colon with its surrounding anatomy [28], as well as less detailed, black-and-white drawings from the informed consent form [29], which also depicted only the colon and the colon with its surrounding area. Additionally, raw, unannotated sample magnetic resonance imaging (MRI) scans [30] were shown. Participants were also shown a rotatable general 3D surface model of the digestive system (see Figure 1A) and the same 3D model projected with AR onto the body (see Figure 1B).

The AR visualization was developed and integrated into a custom-built app on a tablet. Within the app, users positioned themselves in front of the device and viewed themselves through the front camera. The tablet’s camera captures the user and projects an organ model of the digestive system onto their body. The first marker-based prototype, developed with Unity 2021.3 and the EasyAR framework 4.5.0, was used in groups 1 and 2. Participants positioned a paper marker at their abdomen. The app recognized the marker and displayed the organ model accordingly.

The second prototype did not depend on the use of a marker. It was developed with Unity 2022.3 and used the MediaPipe Unity plugin. MediaPipe offers a pose-tracking solution that outputs 3D coordinates for certain body key points from the camera image [31]. The 3D organ model is placed and aligned using the pelvis and shoulder key points. A Samsung Galaxy Tab S6 Lite Android tablet was used for both prototypes.

### Participants and Recruitment

The study was conducted with ad hoc groups in order to minimize the risk of pre-existing relationships influencing the discussion, as pre-existing groups may have established dynamics and be affected by formal or informal hierarchy [22]. Groups were specifically formed to be homogeneous to avoid complications from mixing physicians and patients. This approach promotes stronger identification and unity within the groups while preventing the emergence of hierarchies, as status differences can contribute to inhibited participation and can stifle open discussion [32].

Participants were required to be patients who had previously undergone surgery for colorectal cancer, thereby facilitating their understanding of what information was essential and what they wished they had known about the treatment. Preoperative patients were excluded, because they are difficult to reach due to the worries and anxiety associated with their upcoming surgery.

Patients were identified from the hospital patient database, which included approximately 1300 cases. The database was screened for patients who had undergone surgery within the past 5 years and who were 65 years of age or younger. These candidates were contacted via telephone. The response rate for telephone recruitment was low; only 1 participant agreed. Therefore, patients with Crohn disease were also included in the first group, as the surgical procedures (eg, colectomy and stoma formation) [33,34] and the postoperative experiences (eg, bowel function changes, stoma care, and recovery challenges) are comparable. In addition, patients were recruited through an open participation call published in a local newspaper. Those who expressed interest in the study were provided with the relevant information via email. Additionally, a collaboration was sought with a rehabilitation clinic that houses a colorectal cancer center to create a group of patients currently undergoing rehabilitation. Flyers were placed in the patients’ mailboxes to achieve this.

Inclusion criteria for physicians included regularly providing patient education about colorectal cancer. Physicians were directly engaged by presenting the study during the hospital’s morning meeting and inviting interested physicians to contact the research team to schedule participation.

### Data Analysis

The recordings were transcribed using the content-semantic transcription method [35]. All transcripts were revised and corrected, and the participants’ identities were anonymized during this process. The transcripts were analyzed using the 7-phase framework of content-structuring qualitative content analysis by Kuckartz [36]. In this multistage process, the following main categories were first derived as a priori categories from the thematic categories of the interview guide: personal experiences, information content topics, presentation of content, context of use, and acceptance. The material was initially coded using these main categories, which led to the emergence of an additional main category “features.” Next, the text segments corresponding to each main category were summarized, and subcategories were inductively formed for all main categories except “features,” as no further subdivision could be determined. These categories were discussed by two persons until consensus was reached. The material was coded again using the differentiated categories in a second coding process. All text segments were reviewed again and, based on their level of generality, assigned to subcategories or, when appropriate, to the main categories. The transcription and coding processes were conducted using MAXQDA 2024 [37]. To determine the sentiment regarding the visualization modalities, a sentiment coding scheme was used. Text segments related to each visualization modality were identified and coded on a 5-point scale, ranging from “--” (strongly negative) to “++” (strongly positive), reflecting the overall sentiment expressed by the participants. The evaluation not only took into account the participants’ explicit wishes but also analyzed which aspects of the disease, treatment, and patient education the participants disclosed and expressed interest in. Statements from patients and physicians were considered separately to address the research questions and to account for the distinct needs of these two groups. Technical commitment scores were calculated for each participant by summing the answers on the 1‐5 Likert scale for the 12 items. The mean technical commitment score was then calculated for each group. For smartphone and tablet use, the mean value of the 1‐5 Likert scale was also calculated for each group.

## Results

### Sample Characteristics

A total of 89 patients and 18 physicians were contacted directly to take part in the study, and 27 patients responded to the call for participation in the newspaper. We conducted 5 focus groups (see Table 1) with a total of 30 participants: 4 groups consisted of patients (n=23; 15 women, 8 men) who were previously diagnosed with colorectal cancer, with the exception of 2 participants in group 2 who had Crohn disease, and 1 group consisted of physicians (n=7; 3 women, 4 men).

**Table 1.** Demographic characteristics and questionnaire results by focus group (5 focus groups) for participants (7 physicians and 23 patients) in a qualitative study conducted between July 2022 and September 2023 at a hospital, university, and rehabilitation clinic exploring requirements for a mobile health (mHealth) app for patient education about colorectal cancer.

Members	Focus group number	Participants, n	Age (years), mean (SD)	Gender	Technical commitment score, mean (SD)	Smartphone use (1-5), mean (SD)	Tablet use (1-5), mean (SD)
Physicians	1	7	35.6 (6.3)	3 women, 4 men	43.7 (7.2)	4.17 (0.41)	3.33 (0.82)
Patients (entire patient sample)	2‐5	23	61.0 (11.9)	15 women, 8 men	39.5 (6.3)	4.23 (0.81)	3.56 (0.78)
Patient subset (CRC^a^ and CD^b^)	2	3	57.7 (7.6)	3 women, 0 men	38.3 (11.0)	4.67 (0.58)	3.00 (0)
Patient subset (CRC)	3	4	69.3 (10.8)	2 women, 2 men	41.7 (7.0)	4.33 (0.58)	3.67 (0.58)
Patient subset (CRC)	4	5	67.2 (11.1)	3 women, 2 men	38.8 (6.7)	3.80 (1.10)	3.40 (1.14)
Patient subset (CRC)	5	11	56.9 (12.3)	7 women, 4 men	39.6 (5.5)	4.27 (0.79)	3.75 (0.71)

a CRC: colorectal cancer.

b CD: Crohn disease.

A total of 28 participants completed the questionnaire. The mean technical commitment scores were 39.5 (SD 6.3) for patients and 40.4 (SD 7.2) for physicians.

Of the participants, 96% (27/28) reported using their smartphones at least occasionally (smartphone use≥3). Similarly, most of the physicians (5/6, 83%) and patients (17/22, 77%) also reported using a tablet at least occasionally (tablet use≥3). The frequency of smartphone use exceeded that of tablet use among both groups.

### Category Overview

The analysis identified 23 subcategories organized according to the main categories and were mainly derived from the interview guide (see Table 2). The “features” category was added during the first coding process, reflecting participants’ feedback regarding desired app functionalities. The analysis was structured around the main categories described in the following paragraphs.

**Table 2.** Categories derived from qualitative content analysis of focus group interviews with patients with colorectal cancer and physicians regarding requirements for a mobile health (mHealth) app for patient education.

Main category	Subcategories
Personal experiences^a^	Course of the disease Information-seeking Psychological impact
Information content topics^a^	Surgery risks and complication Treatment alternatives Comprehensibility and technical jargon General information about colorectal cancer Personalization Life consequences and restrictions Perioperative procedure, aftercare Anatomy Treatment procedure
Presentation of content^a^	Visualization modality Interaction Level of detail
Context of use^a^	Timing People and location
Features	N/A^b^
Acceptance^a^	General Data protection Usability, technical complexity Trustworthy, up-to-date content

a A priori categories derived from the interview guide.

b N/A: not applicable.

Within “Personal experiences,” key themes included navigating the course of the disease, reflecting on life consequences, and experiencing psychological impact. “Information content topics” encompassed desired details about surgery risks and complications, treatment alternatives, and understanding colorectal cancer anatomy. “Presentation of content” focused on preferences for visualization modality (2D vs 3D vs AR), desired level of detail, and the importance of interactive features. “Context of use” explored ideal timing for information delivery, preferred locations for access, and the role of family and friends. “Features” included desires for resources like support groups, personalized information, and tools for tracking progress. “Acceptance” encompassed themes of trust in the app, concerns about data privacy, and usability considerations. These categories included all statements, regardless of participant sentiment or differing viewpoints. The results are presented in the following sections, organized by main category.

### Personal Experiences

#### Course of Disease

Patients described their experience with colorectal cancer as a challenging and often lengthy process involving multiple surgeries, complications, long-term adjustments, and setbacks along the way. Many patients underwent multiple surgeries, including the removal of parts of the colon or rectum, creation of a stoma, and later reversal of the stoma.

*And then, in the end, the stoma didn’t help either, unfortunately. And then in May last year, my rectum was amputated. And that was a major operation*.

[P3, group 2]

For some patients, the cancer was not operable or had metastasized, requiring ongoing treatment with chemotherapy, radiation, and/or targeted therapies.

Patients shared insights about the importance of regular colorectal cancer screening, even in the absence of symptoms, as many were diagnosed unexpectedly through routine check-ups or screenings. They emphasized the need for better public awareness and education about colorectal cancer screenings.

Patients felt well cared for and supported by their health care providers, especially when there was good communication, organization, and a sense of being understood. Patients appreciated feeling respected and treated as partners in their care. In contrast, poor communication, long wait times, and insensitive comments from health care staff led to negative experiences and increased anxiety.

#### Information-Seeking

Patients consistently emphasized the importance of being well-informed and having access to detailed information. They appreciated when physicians provided thorough explanations, visual aids like diagrams, and opportunities to ask questions. Patients felt this helped reduce their anxiety and uncertainty and allowed them to be more involved in decision-making.

*The people affected should be given orientation immediately. That takes away a lot of fear*.

[P3, group 3]

This underscores the anxiety-reducing effect of providing patients with adequate information. However, some patients also avoided excessive information, especially if it caused them more distress or fear. They may prefer to focus on the essential details and leave the rest to the physicians. Many reported turning to additional sources like the internet, books, and support groups. However, some cautioned against relying on unverified online sources, which can lead to misinformation and increased anxiety.

*And you don’t even know what sources they used, or if they used any sources at all, or what idiots wrote what. [...] Afterwards, you’re even more uncertain than you already were*.

[P5, group 5]

Overall, patients emphasized the need for a balance: They wanted to be informed but also have the freedom to choose how much information they engaged with at any given time.

*Sometimes you don’t want to know anything about it*.

[P4, group 3]

*You don’t have to look at it. You always have the freedom to choose not to*.

[P3, group 4]

Physicians reported that patients have varying preferences regarding the level of detail they want in medical information. They try to assess each patient’s individual needs and tailor the information accordingly.

*50% of people say: “No, I don’t want to know any of that*.”

[P5, group 1]

#### Psychological Impact

The diagnosis of colorectal cancer and its treatment were often associated with significant emotional distress, including fear, anxiety, and uncertainty. Participants described their initial reaction as feeling shocked and going through a breakdown as they grappled with the news.

*I was scared, scared, and scared. […] I stood in front of the sink and shouted into the mirror: “Dear God, let me live.” And that’s when I had a breakdown, I was sweating and getting really hot, and I just snapped*.

[P1, group 3]

Patients emphasized the importance of psychological support and coping strategies for managing the mental health challenges. They also highlighted the value of counseling or support groups.

### Information Content Topics

#### Surgery Risks and Complications

Patients wanted to be fully informed about the procedure and any potential complications, including specific complications such as wound-related issues like infections or hernias.

*What would be interesting and important to know are the complications that can arise*.

[P3, group 3]

In contrast, physicians reported a divergence in the preferences, with some patients not wanting to know too many details about the risks and complications.

*I would differentiate between those who simply don’t want to know about the risks because they are already so nervous and afraid and those who are genuinely concerned about the risks*.

[P2, group 1]

They recognized the legal requirement to inform patients about risks and complications but also try to provide this information in a less overwhelming manner. This suggests that complex trade-offs exist between legal requirements, patient preferences, and physicians’ expertise.

#### Treatment Alternatives

Clear alternatives to treating colorectal cancer are often limited, as surgery is typically the primary and necessary course of action. Patients may seek second opinions, but physicians often find the treatment path to be straightforward with little room for alternatives.

*With colon cancer, there is no alternative. Either you operate and cure the person, or you don’t*.

[P5, group 1]

#### Comprehensibility and Technical Jargon

Patients often struggled to comprehend medical terminology and jargon used by health care providers and felt overwhelmed by the complexity of the language.

*I received a discharge letter at some point, and it also contained a lot of gibberish. I took a photo and sent it to my sister, who works in cardiology, and asked her what they had done*.

[P1, group 5]

Patients recommended presenting medical information, such as discharge letters, test results, and additional materials, in clear and accessible language, with explanations of technical terms to improve understanding.

Physicians also recognized the importance of simplifying language and avoiding complex medical terminology for patient-friendly communication but also noted the difficulty of balancing clarity with accuracy.

*For example, when we say “stoma,” of course we try to say “artificial bowel outlet,” but often we still use too many technical terms, even when we try hard*.

[P2, group 1]

#### General Information About Colorectal Cancer

Patients were interested in learning more about general information related to colorectal cancer, particularly how it spreads and metastasizes and information about demographics. In general, however, patients wanted more specific, tailored information about their own cancer cases rather than just general cancer facts.

*So that you really understand how it gets from the colon cancer (ie, from the tumor, to the liver, or from the left colon tumor to the right ovary)*.

[P2, group 5]

*I also wanted to know the chances of survival*.

[P1, group 3]

#### Personalization

Patients expressed a desire for information and instructions, such as discharge instructions or dietary guidelines, to be specific to their individual surgery or treatment.

*I would love it if it were possible for the physician to add certain items for the patient*.

[P8, group 5]

*[…] that this is prepared and explained for each individual case*.

[P3, group 3]

This preference for personalized information reflected the desire to understand the unique implications of their diagnosis and treatment, moving beyond a one-size-fits-all approach.

However, physicians were unsure whether tumor location–specific materials are truly necessary for patients. In the case of colorectal cancer, a more standardized approach to patient education materials, rather than highly personalized content, may be sufficient. Pre-made educational materials tailored to the specific tumor location in the colon, such as the ascending, transverse, descending, or rectal parts, would allow for some level of personalization without completely individualizing the content.

*For the colon, it may not be as relevant that it is individually adapted, but for other organs it may be more interesting*.

[P5, group 1]

This highlights a potential challenge in balancing patient preferences for personalization with the practical constraints of the clinical setting.

#### Life Consequences and Restrictions

Patients reported experiencing a range of challenges as a result of their cancer diagnosis and treatment. Specifically, they reported struggling with daily activities, social interactions, and employment due to digestive and bowel issues, requiring the need for ongoing lifestyle adjustments.

*The physical limitations that remain afterwards are significantly greater, leading to an inability to work or even disability*.

[P1, group 3]

Additionally, patients often described experiencing nerve and muscle damage, including neuropathy, muscle weakness, and loss of sensation, resulting from chemotherapy. Many patients also reported feeling physically exhausted and debilitated, even long after treatment. Furthermore, patients had to adapt to significant dietary restrictions and challenges, which limited the types of foods they could consume.

*For me, it was horrible what they had forbidden me to eat*.

[P2, group 2]

Physicians recognized the importance of discussing the long-term consequences of procedures with patients. Patients were concerned about how their treatment would impact their overall quality of life, whether due to a stoma, dietary restrictions, or other functional limitations. They reported that patients wanted to know how these changes will affect their daily routines, relationships, and overall well-being.

*The typical question is about a stoma. That’s always where most patients experience limitations in their quality of life*.

[P6, group 1]

This highlights the need to address not only the immediate medical aspects of cancer treatment but also the wider psychosocial and physical challenges that patients will face in the long term.

#### Perioperative Procedure, Aftercare

Patients emphasized the importance of clear information regarding the perioperative process, including rehabilitation and follow-up care after surgery, with some highlighting the value of dedicated rehabilitation facilities. This indicates that patients want to actively prepare for and manage the challenges of surgery and recovery.

Physicians emphasized the importance of a patient-centered approach throughout the perioperative phase while providing information on the organizational workflows and timelines. The app should also recommend healthy behaviors, such as smoking cessation and mobilization, and enable patient self-monitoring. This demonstrates a recognition of the need for ongoing support and patient engagement throughout the treatment journey.

*[…] from admission to the surgical procedure, how many days patients stay in the clinic, what the goals are after the surgery (ie, freedom from pain, mobilization, post-operative recovery, etc.), and stay in intensive care*.

[P3, group 1]

#### Anatomy

Participants expressed a desire for a basic understanding of human anatomy, such as the path food takes through the body, the functions of different organs, and the relationships between them. Several patients expressed a lack of understanding regarding the different parts of the digestive tract, such as the distinction between the small and large intestine, the function of the appendix, and the location of organs such as the liver and stomach.

*I had no idea about size or the position of organs […] I have always thought, the stomach is horizontal here*.

[P3, group 3]

This illustrates a gap in patients’ preoperative anatomical knowledge, which could potentially hinder their ability to fully understand their diagnosis and treatment options.

Physicians reported providing anatomical explanations before procedures to help patients understand the surgery. These explanations were typically presented on a basic schematic level, focusing on key structures and their general locations.

This highlights the potential benefit of providing patients with accessible, anatomical resources that go beyond the specifics of their operation and support a broader understanding of their condition.

#### Treatment Procedure

Patients have a strong desire for detailed information about the surgery and anatomy involved. They want to know specifics, such as the location and extent of the tumor or affected area, as well as a step-by-step description of the procedure, including how the remaining parts of the colon will be reconnected. Furthermore, patients are interested in seeing “before and after” visualizations to better understand the changes that their body will undergo as a result of the surgery.

*They just said, “I will cut this and that out,” but I actually think it would be quite interesting to see how it’s sewn together*.

[P2, group 5]

This includes information about potential outcomes, such as the possibility of a temporary or permanent stoma.

*Before that, it was about not having a stoma at all […] then he said it could heal better with a temporary stoma. And I went from not at all, to temporary, to ultimately permanent*.

[P10, group 5]

Patients typically focused on the type and extent of their surgical procedures when discussing their treatment, which may include the removal of parts of the colon, the creation of a stoma, or the use of minimally invasive or open surgical techniques. Many also shared their experiences with unexpected complications or additional surgeries, such as infections or bowel obstructions, as well as their experiences with chemotherapy or radiation therapy as part of their treatment plan.

However, physicians reported that patients are more concerned about the consequences of the surgery, such as its impact on their quality of life, rather than the technical aspects of how the surgery is performed.

*It’s not so much about the details of the operation, but rather: What happens postoperatively in the short, medium, and long term? I don’t think how it’s done anatomically is all that relevant for the patient*.

[P5, group 1]

Although patients show limited interest in the specifics of the surgical approach according to the physicians, they often ask about the extent of the resection.

*The most frequent question is: “How many centimeters will be removed?”*

[P6, group 1]

The findings suggest that patients are highly interested in the surgical procedure, whereas physicians tended to perceive patients as less concerned with technical operative details. This suggests that patient education tools should foreground the extent of the resection rather than elaborate on the technical steps of the operation.

### Presentation of Content

#### Visualization Modality

Patients and physicians expressed different preferences for visualization modalities, as shown in Table 3. Key findings revealed positive sentiment toward 3D models (patients: ++, physicians: +) and 2D visualizations (both: +), negative sentiment toward raw MRI scans (patients: -, physicians: --), and mixed to positive sentiment toward AR (patients: +, physicians: ±).

**Table 3.** Patient and physician sentiment toward each visualization modality during focus group interviews, as coded on a 5-point scale ranging from strongly negative (--) to strongly positive (++).

Modality	Patients	Physicians
MRI^a^ (raw, unannotated)	-	--
2D (black and white, colored, only colon, colon+ surrounding)	+	+
3D model	++	+
AR^b^ (marker)+3D model	+	±
AR (markerless)+3D model	+	No data

a MRI: magnetic resonance imaging.

b AR: augmented reality.

Patients and physicians demonstrated different preferences for the visualization modality. Patients tended to find 2D schematic diagrams and illustrations helpful for understanding the anatomy and planned surgical procedures.

*I could do something with that. My bowel, roughly, that’s where they would cut, and that’s what would come out*.

[P1, group 2]

They also found 3D models and animations useful for gaining a better spatial understanding.

*And I would say that it’s much easier to see there than on a piece of paper*.

[P11, group 5]

In contrast, many patients felt that abstract MRI or computed tomography images were too difficult to interpret on their own and they would prefer more user-friendly visualizations.

*I need a lot of imagination to picture where in my body that is*.

[P2, group 2]

Additionally, patients showed interest in and motivation to use AR visualizations, seeing potential for a better understanding and connection to their own body and medical condition. However, several participants expressed doubts about the practical usefulness of the AR visualization, viewing it more as a gimmick than a truly helpful tool. They generally preferred a combination approach, where the AR visualizations were supplemented by more traditional visualizations.

*Oh, wow, awesome. […] Is the liver that big? […] It’s quite nice, but what’s the point of doing it like this, as opposed to the other pictures?*

[P1, group 2]

Physicians tended to favor simple, 2D visualizations over more complex 3D models or medical images. They believed that 3D or interactive visualizations would be too confusing for patients, especially older patients. They viewed AR visualization as a novelty, rather than as a practical tool for their medical practice.

*I mean, it’s a nice gimmick, but what’s the point?*

[P6, group 1]

However, they were open to using short, simple videos to illustrate procedures. These divergent preferences suggest that an mHealth app should offer a range of visualization options. This would allow patients to select the modality best suited to their individual needs and learning styles. It would also provide physicians with visualizations consistent with their practice.

#### Interaction

Interactive features like zooming and enlarging images, cursor- or touch-activated information pop-ups, and the ability to manipulate 3D models to explore different perspectives were desired by patients.

*If you could go there and zoom in.*

[P3, group 3]

*Also, to potentially rotate it, to get a better view of other specific parts, for example*.

[P8, group 5]

Physicians wanted the ability to draw or annotate and interact with the visual aids directly on the app to further explain the surgery. They suggested using digital tools like tablets and styluses to use features like highlighting, circling, and drawing arrows.

*Ideally, you could enter it yourself with a tablet and a pen. Basically, exactly the same as what we’re doing on paper now*.

[P3, group 1]

These findings suggest an app should incorporate interactive elements to cater to different needs. For patients, this would mean providing intuitive controls for exploring visualizations in detail. For physicians, this indicates a preference for digital tools that mirror their established methods of visual communication with patients.

#### Level of Detail

Patients wanted visualizations specific to their own individual anatomy and medical condition rather than generic organ models and wanted to see representations of their own condition. Many wanted very detailed and comprehensive visualizations that show the affected organs as well as the surrounding anatomy.

*I definitely think the surrounding organs need to be included when it comes to the ovaries, or if the liver, stomach or something else is affected, so you can say “it’s here and here*.”

[P4, group 5]

Some patients felt that a more stylized or simplified representation, while less detailed, can still be very helpful in understanding their condition.

However, physicians believed that too much detail and complexity in the visual aids can be overwhelming and confusing. Physicians held mixed views on the level of personalization in the visual aids. Some suggested using a generic, textbook anatomy rather than highly individualized representations. However, others proposed starting with a general overview of the human body then allowing the physician to selectively highlight or hide specific organs or areas of interest.

### Context of Use

#### Timing

Both patients and physicians identified multiple stages of the treatment pathway where an app could be valuable: before the informed consent discussion, to prepare for the upcoming conversation, and clarify available options and resources.

*This app should be presented during the diagnostic consultation, so you can understand what was explained to you during the informed consent discussion*.

[P7, group 5]

*It’s most helpful to give it to them before the informed consent discussion, so they’ve already heard about it beforehand*.

[P6, group 1]

During the informed consent discussion, physicians would use the app to provide information and visuals to the patient. Patients reported that many questions arose only after the discussion or even after surgery. The app could serve as a reference, allowing the patient to review the information in peace and clarify any remaining questions, as they may not have time nor the mental capacity to absorb everything during the acute phase.

*And then, when you’re more or less able to think again, that’s when the questions come*.

[P1, group 2]

Overall, both patients and physicians saw value in the app throughout the entire cancer care journey, from diagnosis to recovery, serving as a resource for both patients and physicians at multiple stages of treatment.

#### People and Location

Patients indicated they would primarily access information and use the app to review the information at home on their own or with their relatives who are often involved in the care process and need to be informed as well. In addition, they wanted to use the app to share information with other health care providers, such as their primary care physician or specialists, to ensure continuity of care.

*To show others, or even other doctors, if, for example, you have problems with the scar afterwards, and then they ask what was done, and then you start telling them again*.

[P4, group 4]

Physicians would use the app during the consultation and indication discussion to provide visual aids and explanations to the patient. This could then be saved in the app for the patient to refer to later.

### Features

In addition to providing core information and educational resources, participants suggested several features that could make the app a more valuable, supportive tool. Patients requested information on resources such as certified cancer treatment centers; rehabilitation facilities; and available social services, benefits, and support resources to which they may be entitled.

*There’s this and that, and of course it would be great if there was a list*.

[P8, group 5]

*Where to find reputable addresses*.

[P4, group 5]

Patients often prepare notes with their questions and concerns before physician visits. To facilitate patient-physician communication, they suggested a feature allowing them to record questions and notes into the app before the visit, which the physician could then review beforehand to be better prepared.

*Where you can enter your questions, where you can enter notes, so that when you get to your next appointment, you can see them*.

[P2, group 2]

Physicians saw potential in using the app to support quality management and continuous improvement of care, such as patient outcomes tracking, and feedback mechanisms.

*What we do verbally during rounds with the pain scale and such, they can also do themselves. This can be visualized with smileys […] in terms of quality management*.

[P3, group 1]

Patients highlighted the value of centralized access to their medical records, including lab results, pathology reports, and surgical details, to ensure continuity of care across multiple health care providers. This highlights the importance of a patient-centered approach to data management that enables individuals to play an active role in their own care.

*If all this paperwork and the visualizations were included in the app, I could really see myself using it*.

[P5, group 4]

### Acceptance

#### Overview

Most participants expressed positive attitudes toward and interest in using an app for patient education. Acceptance of the proposed technology was generally positive across all groups, though some physicians expressed concerns about increased workload for medical staff.

Yes, but who’s going to put that in? Not me, that’s for sure.

[P6, group 1]

This suggests that a successful implementation will require careful consideration of workflow integration and potential administrative burdens.

#### Data Protection

Participants debated the balance between data protection and the acceptance of digital health technologies. Some were concerned about data breaches and misuse, while others viewed data sharing as a way to contribute to research, which highlights the importance of transparent data privacy policies for building trust.

*I’m not so sure about electronic health records, honestly. Look what happened where they were hacked*.

[P1, group 2]

*I am so transparent, I am digitized and stored everywhere, what do I want to hide? I can only contribute to a good cause*.

[P1, group 3]

#### Usability, Technical Complexity

Although some patients were open to the use of technology in health care, others were skeptical and expressed reservations about adopting new technologies.

*If you want to digitize everything and then no longer hand out such paper documents, then that is of course ideal*.

[P1, group 4]

*I won’t enjoy that very much, as I’m not a techie. I’m too old for that*.

[P6, group 5]

Some patients were concerned that they might feel overwhelmed by an app if they don’t know how to use it. There was also concern that an app with too many features might be overloaded.

*Of course, this should not result in users feeling abandoned with the app*.

[P5, group 4]

*If it’s easy to use, older people will be able to handle it too*.

[P1, group 2]

This variability underscores the need for a user-centered design approach that caters to a wide range of digital literacy levels. Simplicity and intuitive navigation are essential for maximizing user adoption and minimizing frustration.

Some physicians advocated for digitization for reasons of environmental friendliness and efficiency, while some expressed concerns that overly complex or digitized solutions could alienate or overwhelm some users, particularly older patients. Some stated that overly complex technology could distract from the main purpose of patient education and understanding. The focus should remain on clear and effective communication.

*Because we live in the 21st century. And because we have everything digitally. Everything is becoming increasingly digitized […] to the “paperless hospital.”*

[P3, group 1]

*I would also keep everything as simple as possible from a technical standpoint. We’re talking about elderly people here*.

[P6, group 1]

#### Trustworthy, Up-to-Date Content

Patients discussed the importance of having access to trustworthy health information, particularly through a reliable app, rather than relying on unverified online sources. Some patients avoided searching for information on the internet because they feared finding unreliable or even “frightening” information.

*I googled it once, and I’ll never do it again*.

[P11, group 5]

There is a clear preference for content that is scientifically based and not driven by commercial interests or profit motives, unlike some websites.

*There is always something that you are supposed to buy or do*.

[P3, group 3]

This underscores the critical importance of providing scientifically sound, evidence-based content within the app, coupled with transparent content curation and ongoing maintenance to ensure continued relevance and user confidence.

## Discussion

### Key Findings

This study used a qualitative approach, using focus group interviews with patients and physicians to identify the essential requirements for an mHealth app designed to support informed decision-making for patients with colorectal cancer, with a specific focus on the potential of AR for visualization. This research explored the patient and physician perspectives on these requirements, particularly regarding information delivery and visualization, to guide app design. Focus group interviews with 23 patients and 7 physicians revealed notable differences in preferences. Participants described current patient education as fragmented, insufficient, and lacking personalization and reported feeling unprepared. They expressed a desire for detailed information about surgery, complications, treatment options, and the impacts on quality of life, with a strong need for personalized content tailored to their specific treatment plan. Physicians expressed a belief that patients are often less interested in in-depth information and prefer a basic overview. Regarding visualization, patients favored interactive 3D models to understand general anatomy and procedures. Physicians preferred simpler, generic 2D images for overviews of the anatomy and procedure that could easily be integrated into their existing workflow and used during informed consent discussions. Participants were engaged by the AR visualization but generally viewed it as a novelty rather than a core component. They expressed a preference for a combination approach where the AR experience supplements more traditional visualizations. Patients envision using such an app at various stages of their treatment journey: as an educational tool to review information and prepare for the informed consent discussion after diagnosis, prepare for treatment, access guidance and track progress during hospitalization, and access information and resources during recovery after treatment. Overall, participants had positive views about a patient education app, emphasizing the importance of user-friendliness, data privacy, and trustworthy content. These findings reveal a discrepancy between patients’ expressed information needs and physicians’ perceptions of those needs, highlighting the importance of directly soliciting and incorporating patient perspectives into app development.

### Interpretations

Patients expressed a lack of understanding of basic anatomy and medical knowledge, a deficit often underestimated by physicians [38]. This finding highlights an issue within health care communication, potentially stemming from time constraints and a reliance on technical jargon. This knowledge gap not only hinders informed decision-making but also can lead to ineffective doctor-patient communication and potentially compromise patient safety [39].

Patients demonstrated a strong desire for detailed information about their condition, such as how the tumor spreads and metastasizes, and information about demographics, consistent with the work of Jane Spalding et al [40] who emphasized that such details are essential for a thorough understanding. This desire for specific information suggests a strategy for managing uncertainty and proactively understanding their illness. Concerns for quality of life and long-term consequences highlight the profound psychological impact of colorectal cancer and the importance of addressing these concerns. O’Connor et al [41] identified a gap in patient satisfaction regarding patient education on the long-term physical and psychological impacts. In our study, patients reported that being informed about their treatment process reduced their anxiety and uncertainty, a finding consistent with the literature [40,42]. Previous studies have reported improved patient understanding with interactive digital patient education interventions [43].

It is important to recognize that patients have varying information needs. Not all patients want detailed information; some prefer a brief overview. These findings are consistent with those reported by Levinson et al [44], who observed that, although nearly all patients want to be offered choices and asked for their opinions, a significant proportion are comfortable leaving final decisions to their physicians and prefer to rely on physician knowledge. These differing preferences likely reflect variations in patients’ levels of health literacy, anxiety about their condition, and preferred coping styles—factors that could be addressed through a personalized app.

Although patients demonstrated engagement with AR visualizations, recognizing their potential to make complex medical concepts more accessible, concerns were raised regarding practical implementation and usability. This suggests a disconnect between the technology’s promise and its current user experience, which likely stems from the fact that the presented AR visualization was a prototype and therefore subject to technical limitations combined with the inherent unfamiliarity of the technology itself. Participants generally viewed AR as a supplementary feature to other visualizations, a perspective that contrasts with research suggesting that such an approach can lead to usability issues [16].

The patient participants exhibited levels of technology commitment similar to those reported in the literature [45]. Despite concerns that older individuals may be overwhelmed by technology, research indicates that they are interested in using mHealth, highlighting the necessity to consider the specific needs and barriers of this target group [46]. The next generation, with more experience using smartphones, may be more comfortable with the technology.

### Implications

This study underscores the need for a patient-centered design approach to mHealth apps for colorectal cancer, prioritizing detailed and accessible information to support informed decision-making and patient empowerment. Specifically, app developers should prioritize features that allow for individualized content delivery, catering to varying levels of health literacy and preferences for level of information detail. An adaptable, patient-centered approach should be applied that allows for individual tailoring of the amount of information as suggested by Edwards [47]. Notably, there is a divergence between the perspectives of physicians and patients on the level of detail that should be provided. This underscores the importance of avoiding assumptions about patient preferences and tailoring information delivery to individual needs.

An app has the potential to serve as a valuable tool throughout the entire patient journey. Specifically, providing it to patients prior to the informed consent discussion appears particularly promising for preparing them for a more informed discussion and allowing them to focus on the essential details and clarify questions without needing to cover basic information first. Poland et al [48] emphasized the importance of incorporating the perioperative phase and the entire patient pathway with timely information and promoting patient engagement. Physicians could use the app to supplement the legally formal information sheet during informed consent discussions.

These findings strongly support the importance of visual aids in patient education. This finding is consistent with research demonstrating that visualizing medical information enhances comprehension of written materials [49]. However, our study revealed differing preferences, suggesting that an app should offer a range of visualization options to satisfy patient preferences while also providing physicians with accessible visuals compatible with their current practice. Future research should investigate the feasibility and usability of AR features in clinical settings. Specifically, studies should investigate the potential of an AR mirror feature to enhance patient understanding and engagement throughout the patient journey, particularly during informed consent discussions. Given patients reported a stronger connection to their bodies when using AR, further studies should explore whether AR visualizations improve their ability to accurately map anatomical structures onto their own bodies, potentially enhancing overall comprehension.

Successful implementation requires careful attention to usability, interface design, data privacy, and security [50]. These factors are essential for ensuring the app is accessible, is trustworthy, and effectively meets the needs of a diverse patient population, ultimately contributing to more informed and empowered patients navigating the life-altering challenges of colorectal cancer.

### Limitations

One of the primary limitations of this study is the participant cohort. The focus groups, comprised of patients who expressed an interest in patient education, may not be representative of the average patient population. This potential bias is due to the self-selection of participants, who may have a higher level of engagement and interest, thereby limiting the generalizability of the findings to the broader patient population. Additionally, the retrospective nature of the study may have introduced recall bias, with treatments occurring from a few weeks to several years ago. This study was limited by the relatively small sample size (n=30) and the focus on patients with colorectal cancer. Therefore, caution should be exercised when extrapolating these results to other cancer types or patient populations. Finally, the participants in the study were younger than the typical cohort presenting with colorectal cancer, whose median age is generally older than 70 years [51]. To improve generalizability and validity, future studies should consider interviewing patients currently undergoing treatment. Furthermore, the study’s reliance on physicians from a single hospital may also limit its generalizability.

### Conclusions

This study found that patients with colorectal cancer require detailed, personalized, and visual information about their condition, treatment, and potential complications. Although physicians sometimes underestimate patients’ need for information, they recognize the challenges that patients face in comprehending the information provided. The ideal approach for a patient education app would combine comprehensible detailed content with interactive customizable visualizations. Patients appreciated interactive 3D models and AR visualizations, which can introduce complex medical concepts in an accessible and engaging way. However, concerns remain regarding practical implementation and usability. Patients preferred to access the app at various points in their treatment journey. This study highlights the crucial role of a patient-centered approach when designing mHealth apps and emphasizes the potential benefits of including AR visualizations as a supplementary feature to enhance patient engagement and understanding.

## Supplementary material

10.2196/75972 Multimedia Appendix 1 Interview guide used in focus groups with patients and physicians.

10.2196/75972 Multimedia Appendix 2 Questionnaire used in the focus groups assessing smartphone and tablet use and general demographic information.

## Abbreviations

AR: augmented reality
CT: computed tomography
mHealth: mobile health
MRI: magnetic resonance imaging
