# Supplementary material for: Requirements for mHealth and Augmented Reality Apps for Patient Education Regarding Colorectal Cancer Surgery: Focus Group Study
Source: JMIR Form Res. 2026 Feb 26;10:e75972. doi: 10.2196/75972 (PMC12945352; doi:10.2196/75972)
Supplement: Multimedia Appendix 1 [file formative-v10-e75972-s001.pdf]

Interview guide

An interview guide was employed, with modifications made to adapt it to patients' and physicians' perspective. All changes in the guide for physicians in adaptation to the guide for patients have been highlighted. Questions that were only asked of patients and not of physicians were crossed out.

Patients

Category	Questions
Welcome	
Personal experiences	Would you like to tell us about the course of the illness, the diagnosis, and the treatment?<br><br>Think of the informed consent discussion you had before colon cancer surgery. Can you describe how these discussions were conducted?
Information content topics	What information did you receive during the informed consent process?<br><br>What topics would you have liked to receive more information about?<br><br>If there were a smartphone or tablet app where you could view relevant information about the treatment, what content should this app have?
Visualization	How was your treatment visualized by the physician?<br><br>Can you describe your experience with the visualization used?<br><br>*Presentation of different visualizations and discussion of pros and cons.*<br>• raw, unannotated sample MRI scans<br>• 2D image of only colon, stylized (black and white)<br>• 2D image of only colon and surrounding, stylized (black and white)<br>• 2D image of only colon, more detailed (colored)<br>• 2D image of only colon and surrounding, more detailed (colored)<br>• 3D model of digestive system presented on a laptop<br>• Augmented reality prototype<br><br>*Discussion on preferred visualization type given these stimuli:*<br>• Text vs. images<br>• Paper vs. digital<br>• 2D images vs. 3D models<br>• Only Affected area vs. also surrounding area<br>• Stylized visualization vs. realistic visualization
Context of use	In what environment and setting could the app be used?
Acceptance	What are the potential benefits and drawbacks of using a smartphone or tablet app for patient education?<br><br>What factors would you make more likely or less likely to use such an app?
Final question	Are there any other comments you would like to add?

Physicians

Category	Questions
Welcome	
Personal experiences	~~Would you like to tell us about the course of the illness, the diagnosis, and the treatment?~~<br><br>Think of the informed consent discussion you do before colon cancer surgery. Can you describe how these discussions are conducted?
Information content topics	What information do you give the patient during the informed consent process?<br><br>What topics do the patients need to receive more information about?<br><br>If there were a smartphone or tablet app where patients could view relevant information about the treatment, what content should this app have?
Visualization	How do you visualize the treatment for the patient?<br><br>Can you describe your experience with the visualization used?<br><br>*Presentation of different visualizations and discussion of pros and cons.*<br>• raw, unannotated sample MRI scans<br>• 2D image of only colon, stylized (black and white)<br>• 2D image of only colon and surrounding, stylized (black and white)<br>• 2D image of only colon, more detailed (colored)<br>• 2D image of only colon and surrounding, more detailed (colored)<br>• 3D model of digestive system presented on a laptop<br>• Augmented reality prototype<br><br>*Discussion on preferred visualization type given these stimuli:*<br>• Text vs. images<br>• Paper vs. digital<br>• 2D images vs. 3D models<br>• Only Affected area vs. also surrounding area<br>• Stylized visualization vs. realistic visualization
Context of use	In what environment and setting could the app be used?
Acceptance	What are the potential benefits and drawbacks of using a smartphone or tablet app for patient education?<br><br>What factors would you make more likely or less likely to use such an app?
Final question	Are there any other comments you would like to add?
