# Supplementary material for: Requirements for mHealth and Augmented Reality Apps for Patient Education Regarding Colorectal Cancer Surgery: Focus Group Study
Source: JMIR Form Res. 2026 Feb 26;10:e75972. doi: 10.2196/75972 (PMC12945352; doi:10.2196/75972)
Supplement: Multimedia Appendix 2 [file formative-v10-e75972-s002.pdf]

Carl von Ossietzky Universität Oldenburg

Questionnaire focus groups

Technology usage

I use my smartphone in everyday life... (choose one option)

☐ always (5) ☐ often (4) ☐ occasionally (3) ☐ rarely (2) ☐ never (1)

☐ I don't own a smartphone

I use my tablet in everyday life... (choose one option)

☐ always (5) ☐ often (4) ☐ occasionally (3) ☐ rarely (2) ☐ never (1)

☐ I don't own a tablet

Additional information

Age in years

Gender

☐ female ☐ male ☐ divers ☐ don't want to answer

Job title (physicians only)

Additional comments
